# GABA_A_ Receptor-Mediated Acceleration of Aging-Associated Memory Decline in APP/PS1 Mice and Its Pharmacological Treatment by Picrotoxin

**DOI:** 10.1371/journal.pone.0003029

**Published:** 2008-08-21

**Authors:** Yuji Yoshiike, Tetsuya Kimura, Shunji Yamashita, Hiroyuki Furudate, Tatsuya Mizoroki, Miyuki Murayama, Akihiko Takashima

**Affiliations:** 1 Laboratory for Alzheimer's Disease, RIKEN Brain Science Institute, Wako-shi, Saitama, Japan; 2 Laboratory of Endocrinology and Neuro-ethology, Department of Regulation Biology, Faculty of Science, Saitama University, Saitama-shi, Saitama, Japan; Mental Health Research Institute of Victoria, Australia

## Abstract

Advanced age and mutations in the genes encoding amyloid precursor protein (APP) and presenilin (PS1) are two serious risk factors for Alzheimer's disease (AD). Finding common pathogenic changes originating from these risks may lead to a new therapeutic strategy. We observed a decline in memory performance and reduction in hippocampal long-term potentiation (LTP) in both mature adult (9–15 months) transgenic APP/PS1 mice and old (19–25 months) non-transgenic (nonTg) mice. By contrast, in the presence of bicuculline, a GABA_A_ receptor antagonist, LTP in adult APP/PS1 mice and old nonTg mice was larger than that in adult nonTg mice. The increased LTP levels in bicuculline-treated slices suggested that GABA_A_ receptor-mediated inhibition in adult APP/PS1 and old nonTg mice was upregulated. Assuming that enhanced inhibition of LTP mediates memory decline in APP/PS1 mice, we rescued memory deficits in adult APP/PS1 mice by treating them with another GABA_A_ receptor antagonist, picrotoxin (PTX), at a non-epileptic dose for 10 days. Among the saline vehicle-treated groups, substantially higher levels of synaptic proteins such as GABA_A_ receptor α1 subunit, PSD95, and NR2B were observed in APP/PS1 mice than in nonTg control mice. This difference was insignificant among PTX-treated groups, suggesting that memory decline in APP/PS1 mice may result from changes in synaptic protein levels through homeostatic mechanisms. Several independent studies reported previously in aged rodents both an increased level of GABA_A_ receptor α1 subunit and improvement of cognitive functions by long term GABA_A_ receptor antagonist treatment. Therefore, reduced LTP linked to enhanced GABA_A_ receptor-mediated inhibition may be triggered by aging and may be accelerated by familial AD-linked gene products like Aβ and mutant PS1, leading to cognitive decline that is pharmacologically treatable at least at this stage of disease progression in mice.

## Introduction

Both aging and mutations in genes that encode amyloid precursor protein (APP) and presenilin (PS) are considered to be major risk factors for Alzheimer's disease (AD). Aß that is generated from APP, partly through the proteolytic action of PS1 complex, oligomerizes and causes neurotoxicity via synaptic dysfunction [1]–[3]; and the appearance of Aß oligomers correlates with the initiation of memory impairment in a mouse model of AD [4]. On average, cognitive abilities decline with age, yet a recognizable subpopulation of older individuals maintains mental abilities [5]. Both aging and gene risks are known to be tightly associated with glutamate excitotoxicity via calcium dysregulation [6]–[8]. On the other hand, the complex symptoms of AD and their partial correlation with pathological hallmarks indicate that understanding the pathogenesis of AD may require investigations of abnormalities at multiple levels; this would enhance our awareness that various compensatory mechanisms are at work in maintaining brain functions [9]. It is desirable to find a way to improve memory based on the common mechanisms responsible for cognitive decline due to aging (i.e., non-genetic) and genetic risks. In the present study, we investigated spatial memory performance and hippocampal long-term potentiation (LTP) in relatively young but mature “adult” (9–15 months) and aged or “old” (19–25 months) non-transgenic (nonTg) mice and in adult APPswe/PS1Δexon9 (APP/PS1) transgenic mice [10]. On the basis of changes we observed in memory performance and LTP linked with enhanced GABA_A_ receptor-mediated inhibition, we tested the effects of picrotoxin (PTX), a GABA_A_ receptor antagonist and observed that 10 days of PTX treatment improves the cognitive functions of adult APP/PS1 mice.

## Results

### Cognitive decline in adult APP/PS1 mice

**Figure 1:**
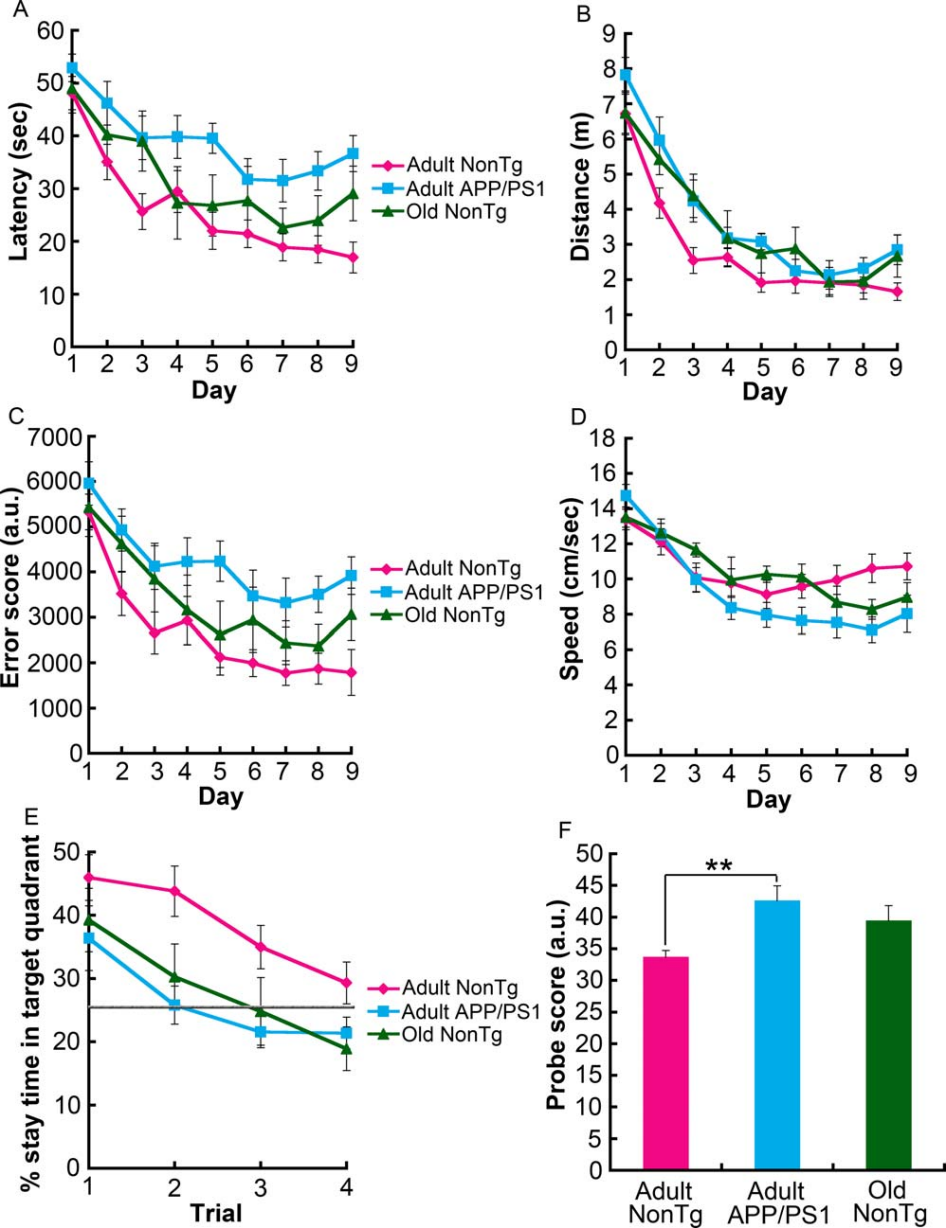
Memory decline in adult APP/PS1 mice in the MWM. (A) Adult APP/PS1 mice, but not old nonTg mice, took significantly longer than adult nonTg mice to reach the hidden platform over the 9-day task acquisition period (F[1,25] = 17.95, p = 0.0003, repeated measures two-way ANOVA). Differences between adult nonTg and adult APP/PS1 mice was also observed in both the distance or the path-length that the mouse swam (F[1,25] = 11.69, p = 0.0022) (B) and in the error score (F[1,25] = 11.79, p = 0.0021), which is defined in the Materials and Methods (C). (D) In terms of swimming speed, no statistical difference was detected among the three groups. (E) Four post-acquisition probe trials were run, and the average percentage of time spent in the target quadrant was plotted. Repeated measures two-way ANOVA analysis showed that adult nonTg mice spent a greater percentage of time searching the target quadrant than adult APP/PS1 mice (F[1,25] = 11.39, p = 0.0024) and old nonTg mice (F[1,19] = 4.56, p = 0.0459). The gray horizontal line in (E) represents random search. (F) The probe scores of adult APP/PS1 mice were significantly higher than those of adult nonTg mice (**p = 0.0033; two-tailed Mann-Whitney U test), suggesting that adult APP/PS1 mice have reduced memory accuracy. The number of mice tested was 13 adult nonTg (9–13 months, 11.0±1.4 months), 14 adult APP/PS1 (9–13 months, 11.0±1.5 months), and 8 old nonTg mice (19–21 months, 20.3±1.2 months). The nonTg mice used in these experiments were wild-type littermates and controls of the single APP/PS1 transgenic line.

The Morris water maze (MWM) test was administered as described in the Materials and Methods. Three groups of male mice from the same line were tested: adult nonTg, adult APP/PS1, and old nonTg. In normal mice, nine days of training in this spatial memory task leads to incremental learning of a constant platform location, and by the 10th day, formation of long-lasting spatial reference memory is clearly manifested [11]. Consistent with previous observations [12]–[14], during the acquisition phase adult APP/PS1 mice took longer to reach the hidden platform than did adult nonTg littermate control mice (F[1,25] = 17.95, p = 0.0003; repeated measures two-way ANOVA) (Figure 1A). Such a substantial delay in learning was not observed in old nonTg mice. These results were recapitulated by two other measures, namely, distance (F[1,25] = 11.69, p = 0.0022) and error score (F[1,25] = 11.79, p = 0.0021) (Figure 1B and 1C). Distance is the path-length that a mouse swims during each trial. The error score is similar to “search error” or “behavioral index,” two parameters described previously [5], [15]. The error score was calculated by integrating the distances between the mouse and platform every 0.5 sec in each trial. The swimming speed did not differ significantly among the three groups (Figure 1D). For the probe tests on the 10th day, the percentage of dwell time spent in the target quadrant was calculated (Figure 1E). Adult nonTg mice had a significant place preference for the target quadrant according to the Friedman test (p = 0.0001). Although adult APP/PS1 mice showed a place preference in the first probe trial (p = 0.0066; Friedman test), by the second probe trial they were clearly searching randomly, since they did not exceed 25% dwell time in the target quadrant, suggesting that even any place preference they acquired quickly became extinct. Old nonTg mice used in this MWM test were wild-type controls from the same lines as the adult nonTg and adult APP/PS1 mice. Statistically, this small cohort from a single line did not show a place preference for the target quadrant during the first probe trial. By repeating the probe test four times, we observed that adult nonTg mice spent more time in the target quadrant than either adult APP/PS1 (F[1,25] = 11.39, p = 0.0024; repeated measures two-way ANOVA) or old nonTg mice (F[1,19] = 4.56, p = 0.0459; repeated measures two-way ANOVA). There was also a significant difference among the three groups according to the first trial's probe score (p = 0.0094; Kruskal-Wallis test), that is, the probe test version of the error score divided by the total number of recorded frames (a frame/0.5 sec×60 sec = 120 frames). A direct comparison also showed a significant difference between adult nonTg and adult APP/PS1 mice (**p = 0.0033; two-tailed Mann-Whitney U test), indicating that the memory of adult APP/PS1 mice was less accurate, since higher probe scores mean that a mouse was swimming in an area distant from the target (i.e., the center of the position where the platform was located during the acquisition period). These results suggest that some of the cognitive functions in APP/PS1 mice decline during adulthood.

### Age-related cognitive decline in wild-type mice

**Figure 2:**
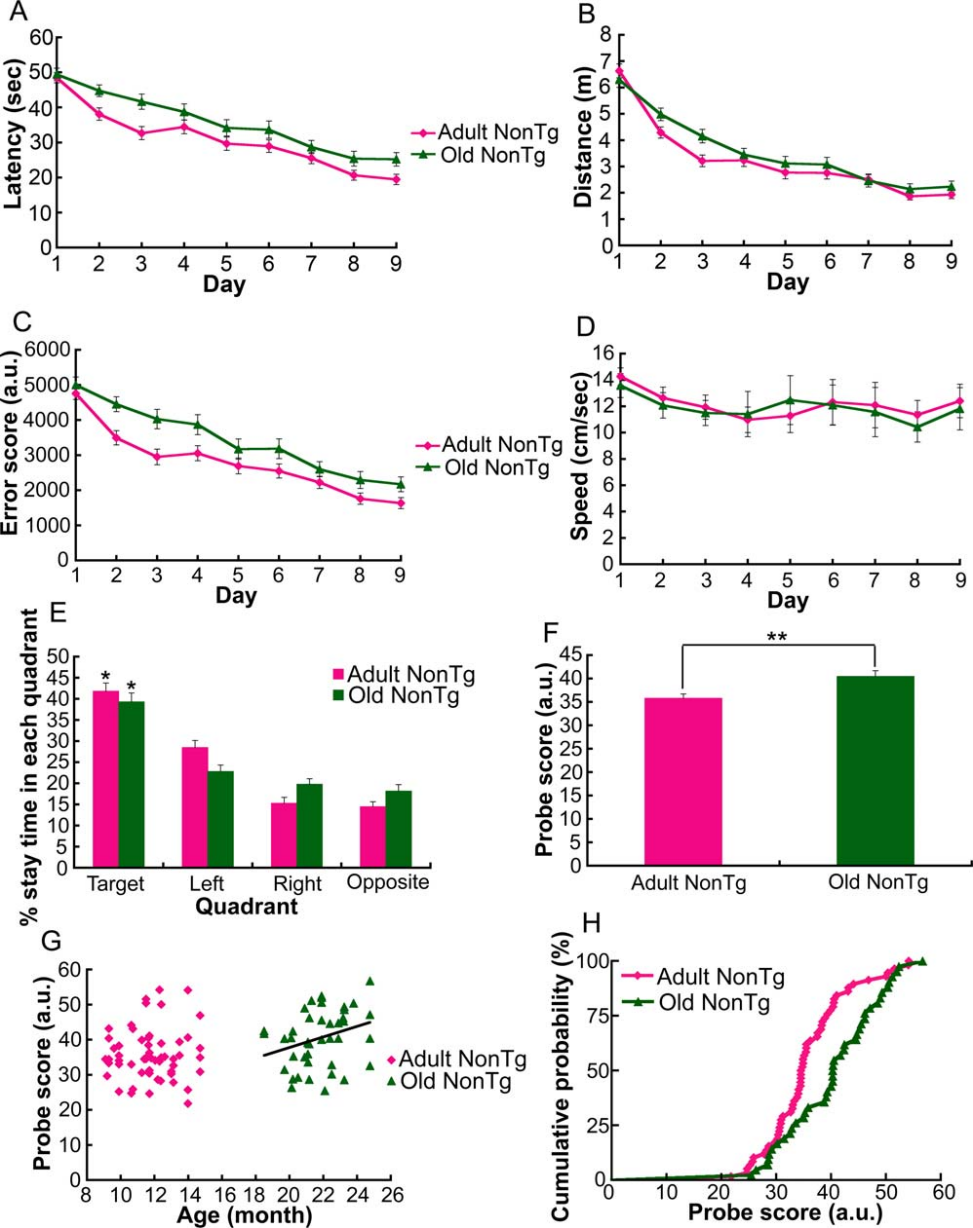
Age-dependent delay in learning and reduced accuracy of memory in wild-type mice. (A) Old nonTg mice took significantly longer than adult nonTg mice to reach the hidden platform (F[1,98] = 5.74, p = 0.0167; repeated measures two-way ANOVA). (B) There was no statistical difference in the distances swam by mice of the two age groups. (C) The error scores of old nonTg mice were significantly higher than those of adult nonTg mice (F[1,98] = 11.42, p = 0.0008; repeated measures two-way ANOVA). (D) The swimming speed of mice of the two age groups was not significantly different. (E) Both adult and old nonTg mice displayed a place preference for the target quadrant in the probe test (both age groups, *p<0.0001; Friedman test). There was no statistical difference in the percentage dwell time in the target quadrant of adult and old nonTg mice. (F) However, the probe scores of the old nonTg mice were significantly higher than those of adult nonTg mice (**p = 0.0038; two-tailed Mann-Whitney U test), indicating that the memory of old nonTg mice was less accurate than that of adult nonTg mice. In terms of both percentage of target-quadrant dwell time and probe score, there was no significant difference between the small cohorts of nonTg mice from the single mouse line used in experiments for Figure 1 and the large cohorts from multiple lines used here at each age group. A significant difference in probe score between the large cohort of adult nonTg mice (n = 58) and the small cohort of adult APP/PS1 mice (n = 14) was ascertained (p = 0.0059; two-tailed Mann-Whitney U test). (G) There was a significant correlation between age and probe score within the old nonTg mouse group (p = 0.0455; two-tailed Spearman test) but not within the adult nonTg mouse group. No correlation was observed among the adult APP/PS1 mice used for the experiments of Figure 1. (H) In the cumulative probability plot, there was also a significant difference in the distribution of probe scores of nonTg mice for two age groups (p = 0.0095; two-sample Kolmogorov-Smirnov test). Mice used in these experiments were nonTg controls from a variety of transgenic lines with a common C57BL/6J background strain. We used 58 adult mice (9–15 months, 11.7±1.6 months) and 42 old mice (19–25 months, 21.8±1.6 months).

Cognitive abilities are known to decline, on average, with age even though a subpopulation of older individuals maintains mental abilities [5]. Thus, we hypothesized that a greater number of mice might show an age-dependent change in cognitive function. Because only a limited number of animals, especially aged ones, were available from a single line, we examined 100 wild-type control mice (58 adult and 42 old) from multiple transgenic lines having in common the C57BL/6J background strain. In contrast to adult nonTg mice, old nonTg mice showed delayed learning performance in terms of latency (F[1,98] = 5.74, p = 0.0167; repeated measures two-way ANOVA) and error score score (F[1,98] = 11.42, p = 0.0008; repeated measures two-way ANOVA) [5], [15], but not in terms of distance (Figure 2A, 2B, and 2C). Swimming speed was not statistically different between adult and old nonTg mice (Figure 2D). Both nonTg mouse groups formed a place preference for the target quadrant in the probe test (both age groups, *p<0.0001; Friedman test) (Figure 2E). However, the probe score of old nonTg mice was significantly higher than that of adult nonTg mice (**p = 0.0038; two-tailed Mann-Whitney U test) (Figure 2F). This result suggests that aged animals memorized the target position less accurately than younger ones. We also analyzed each age group to determine whether the accuracy of memory was age-dependent (Figure 2G). As the trend line indicates in Figure 2G, a significant correlation between age and probe score was observed only in the old nonTg group (p = 0.0455; two-tailed Spearman test), supporting the hypothesis that an age-dependent reduction in the accuracy of memory becomes more prominent in older individuals. We also detected a difference in the distribution pattern of the probe scores of adult and old nonTg mice (p = 0.0095; two-sample Kolmogorov-Smirnov test) (Figure 2H). Whether such a difference in the distribution of a cognitive function also exists in APP/PS1 transgenic mice may be revealed in the future by examining a greater number of animals. The present results suggest that at least, on average, slow learning and inaccurate memory are common features of cognitive decline in adult APP/PS1 mice and old nonTg mice.

### LTP reduction is linked to upregulated inhibition *via* GABA_A_ receptors in adult APP/PS1 and old nonTg mice

**Figure 3:**
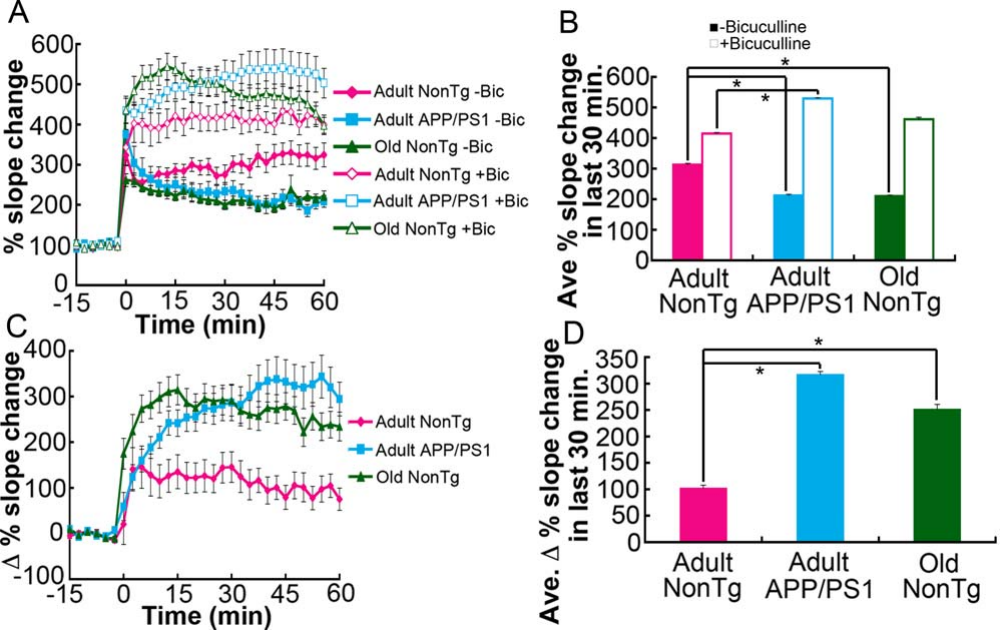
Enhanced GABA_A_ receptor-mediated inhibition reduces LTP in adult APP/PS1 and old nonTg mice. (A) LTP in the molecular layer of the dentate gyrus was recorded in the absence and presence of bicuculline to estimate the extent of GABA_A_ receptor-mediated inhibition. In aCSF-bathed slices, tetanus-induced LTP expression in adult APP/PS1 and old nonTg mice was smaller than that in slices from adult nonTg mice. In contrast, in the presence of bicuculline, LTP in slices from adult APP/PS1 and old nonTg mice was larger than that in slices from adult nonTg mice. (B) Data collected from the last 30 min in (A) were averaged. The means of the three groups in each condition with or without bicuculline showed significant differences in the Friedman test (p<0.0001). However, the Dunn's multiple comparison test (*p<0.001) showed a significant difference between adult nonTg and adult APP/PS1 but not between adult nonTg and old nonTg in the presence of bicuculline. (C) The averaged data in the condition without bicuculline was subtracted from the data in the condition with bicuculline, showing the higher extent of inhibition mediated by GABA_A_ receptors in adult APP/PS1 and old nonTg mice. (D) The values from the last 30 min in (C) were averaged; *p<0.0001; two-tailed Mann-Whitney U test. The numbers of slices and mice used in each condition were as follows: bicuculline-treated slices–17 slices from 9 adult nonTg mice, 24 slices from 9 adult APP/PS1 mice, and 20 slices from 5 old nonTg mice; untreated slices–13 slices from 9 adult nonTg mice, 16 slices from 9 adult APP/PS1 mice, and 22 slices from 5 old nonTg mice. The ages of mice were 9–11 months (9.3±0.7 months) for adult nonTg; 9–11 months (9.5±0.9 months) for adult APP/PS1; and 20–24 months (22.7±2.0 months) for old nonTg mice. The nonTg mice used for LTP measurements were nonTg littermates and controls from a variety of transgenic lines, all having in common the C57BL/6J background strain.

To understand how aging-associated memory decline and memory decline associated with mutant APP/PS1 genes might be linked with changes in the plasticity of synapses, we next measured LTP in the dentate gyrus region of hippocampal slices. *In vitro* slices were prepared from the same three groups of mice. Tetanic stimulation was used to elicit LTP by activating NMDA receptors, thus engaging calcium-dependent processes [16]. Slices from adult APP/PS1 mice and old nonTg mice showed significantly less tetanus-induced LTP compared to slices from adult nonTg mice (Figure 3A and 3B). These results are consistent with results from the MWM tests (Figure 1 and 2), which showed a decline in the cognitive performance of adult APP/PS1 and old nonTg mice.

Deficits in synaptic plasticity have been identified in aged animals with cognitive impairment [17]. Other studies using rodents have also shown that the amount of GABA_A_ receptors increases in the hippocampus with aging [18]–[21]. Therefore, we hypothesized that reduced LTP in slices from old nonTg mice may be related to enhanced inhibition resulting from an increased number of GABA_A_ receptors. To estimate the degree of LTP reduction via GABA_A_ receptors, we measured LTP in the presence of bicuculline, a GABA_A_ receptor antagonist (Figure 3A and 3B). Under this condition, LTP in slices from both old nonTg mice and adult APP/PS1 mice was enhanced compared to LTP in slices from adult nonTg mice. A greater magnitude of LTP in the presence of bicuculline indicated that synaptic inhibition mediated by GABA_A_ receptors in adult APP/PS1 and old nonTg mice was upregulated, perhaps as a possible compensatory response to increased synaptic excitation (Figure 3B, 3C and 3D). Thus, adult APP/PS1 mice and old nonTg mice shared the following characteristics: upregulated GABA_A_ receptor-mediated inhibition linked to LTP reduction and memory decline. Assuming that upregulation of GABA_A_ receptor-mediated inhibition, which usually occurs with aging, was accelerated in APP/PS1 mice, we next examined whether a treatment aimed at normalizing inhibition might improve memory in adult APP/PS1 mice.

### PTX treatment improves memory in adult APP/PS1 mice

Excessive GABA-mediated inhibition was reported in the Ts65Dn mouse model of Down syndrome (DS), which expresses three copies of chromosome 16 genes like APP, GluR5, etc. [22]. It has also been reported that chronic treatment of Ts65Dn mice with the non-competitive GABA_A_ receptor antagonist PTX “at a non-epileptic dose” improves Ts65Dn object recognition memory [23]. AD and DS share many common features, and all individuals with DS develop the neuropathology of AD [24].

**Figure 4:**
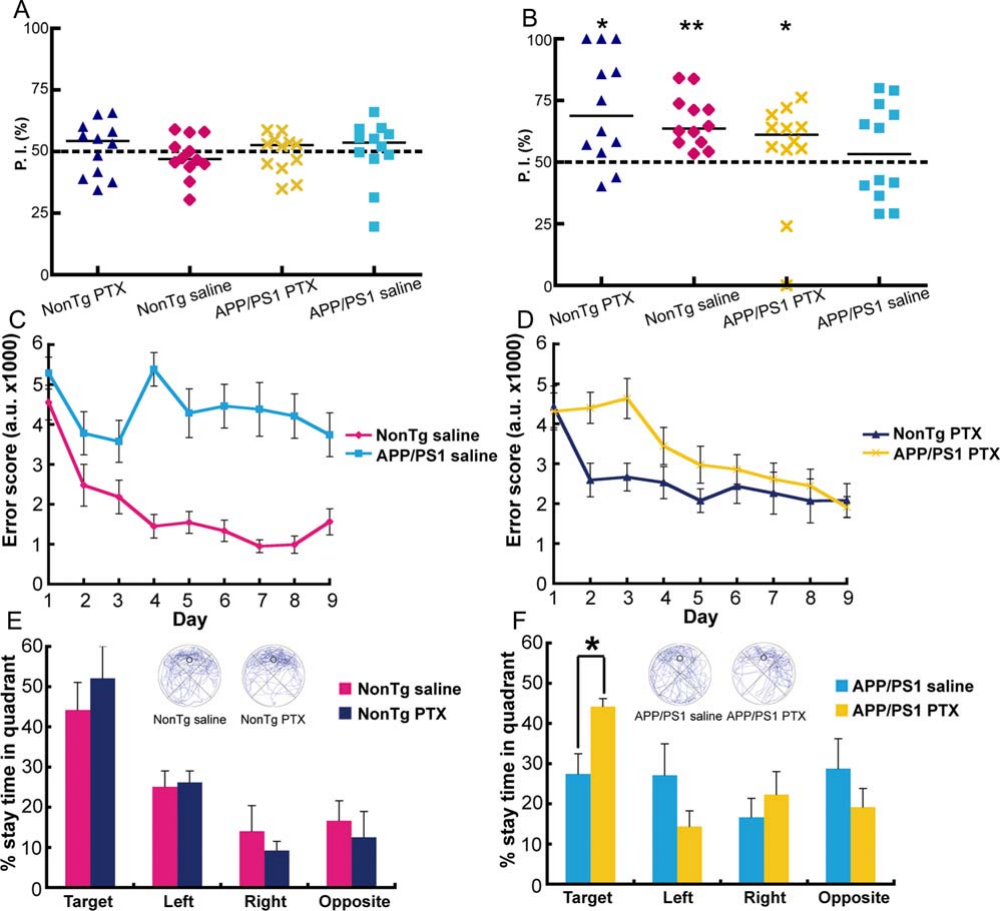
Memory facilitation in adult APP/PS1 mice treated with PTX. The effect of 10-day PTX treatment (at a non-epileptic dose) on short-term object recognition memory was tested with a NOR task. Long-term spatial reference memory was tested with the MWM task. (A, B) Preference indices (P. I.) during training (A) and testing (B) in the NOR task in PTX- and saline-treated nonTg and APP/PS1 mice (n = 6 per group). Adult APP/PS1 mice showed partial recovery of object recognition memory with PTX treatment; *p = 0.0161 and **p = 0.000244, binomial analysis for accessing novel objects. (C, D) Learning session error scores of saline-treated nonTg and APP/PS1 mice (C) and PTX-treated nonTg and APP/PS1 mice (D) tested on the MWM task. In saline-treated groups, adult APP/PS1 mice showed spatial learning impairment (F[1,10] = 13.73, p = 0.0041; repeated measures two-way ANOVA). In PTX-treated groups, there was no significant difference between APP/PS1 and nonTg mice (F[1,10] = 3.08, p = 0.1099; repeated measures two-way ANOVA). (E, F) Performance during the probe trial showed significant improvement in PTX-treated APP/PS1 mice (preference in target quadrant over other quadrants; p = 0.0057; the Friedman test). The swimming traces of the mice are shown in blue. The target quadrant is indicated by a circle, which represents the location of the platform during training; *p = 0.0260; two-tailed Mann Whitney U test. The ages of mice were 9–13 months (10.9±1.6 months) for nonTg saline; 9–10 months (9.9±0.1 months) for nonTg PTX; 9–13 months (11.1±1.7 months) for APP/PS1 saline; and 9–13 months (10.8±1.7 months) for APP/PS1 PTX mice. The nonTg mice used in the drug-treatment experiments were wild-type littermates and controls of the APP/PS1 line.

Since we observed enhanced inhibition in adult APP/PS1 mice, we hypothesized that PTX treatment might also improve memory in this mouse model of AD. Adult APP/PS1 and adult nonTg mice were given daily injections of PTX (1.0 mg/kg in a volume of 10.0 ml/kg) or saline vehicle intraperitoreally in their home cages for 10 days. Then, four groups of adult male mice (saline-treated nonTg, saline-treated APP/PS1, PTX-treated nonTg, and PTX-treated APP/PS1) were tested on a novel object recognition (NOR) task capable of detecting hippocampal and parahippocampal dysfunction in declarative memory processing [23]. After being habituated in a new cage, each mouse was exposed to two identical objects placed in two corners of the apparatus during a 15-min training session (Figure S1). As expected, the four groups of mice explored the two identical objects to the same extent (Figure 4A). After the training session, a 15-min testing session was conducted by replacing one of the familiar objects with a novel object (Figure 4B). Binomial analysis showed that nonTg and PTX-treated APP/PS1 mice preferentially explored the novel object (Figure 4B). In contrast, the group of saline-treated APP/PS1 mice explored novel and familiar objects to the same extent or indiscriminately (Figure 4B). Taken together, these results showed that PTX treatment partially rescued object recognition memory in adult APP/PS1 mice.

Four groups of mice (saline-treated nonTg, saline-treated APP/PS1, PTX-treated nonTg, and PTX-treated APP/PS1) were also tested on the MWM test. Here, we used error score as a measure of learning performance [5], [15]. Saline-treated APP/PS1 mice showed a significantly higher error score than saline-treated nonTg mice (F[1,10] = 13.73, p = 0.0041; repeated measures two-way ANOVA), indicating impaired spatial learning in saline-treated APP/PS1 mice (Figure 4C). When the mice were treated with PTX, no significant difference was observed between the two genotypes (Figure 4D). This result suggests that adult APP/PS1 and adult nonTg mice have similar learning abilities as a result of the PTX treatment. The improvement of learning was directly reflected in their place preference in the probe test (Figure 4E and 4F). Saline-treated APP/PS1 mice searched each quadrant randomly (p = 0.772; Friedman test). The cognitive performance of saline-treated APP/PS1 mice was unlike that of untreated adult APP/PS1 mice, which exhibited a small but significant place preference for the target quadrant (Figure 1E and 4F). We assumed that the complete loss of spatial reference memory in saline-treated APP/PS1 mice was due to stress induced by the daily intraperitoreal injections they received for 10 days. Indeed, the cognitive function of another AD mouse model (APP_V717I_-CT100) has been previously reported to be especially vulnerable to chronic stress [25]. Since it is now widely accepted that an enriched environment improves cognitive function of some transgenic mice, including APP/PS1 [26], [27], it is expected that a poor environment, such as stress from daily injections, would have an opposite effect, causing cognitive function to decline. Most notably, even under these stressful conditions, PTX-treated APP/PS1 mice preferentially searched the target quadrant (p = 0.0057; Friedman test) (Figure 4F). In fact, PTX treatment substantially raised place preference in adult APP/PS1 mice up to the level of adult nonTg mice (Figure 4E and 4F). These results suggested that spatial reference memory in adult APP/PS1 mice was rescued by the 10-day PTX treatment.

### Modification of synaptic protein levels by 10-day PTX treatment

**Figure 5:**
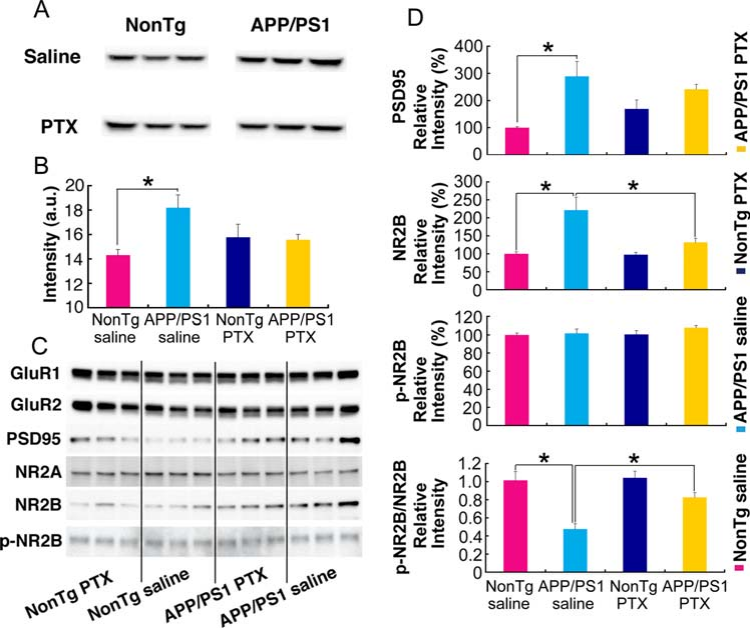
Modification of synaptic protein levels in adult APP/PS1 mice by PTX treatment. The effect of 10-day PTX treatment on the protein level of GABA_A_ receptor α1 subunit was examined by Western blot analysis of brain homogenates. (A, B) Immunoreactivity against the GABA_A_ receptor α1 subunit, as demonstrated by Western blotting, showed that the excess amount of this subunit in saline-treated APP/PS1 mice in comparison with the amount in saline-treated nonTg mice was absent in PTX-treated animals; *p = 0.0274, two-tailed Student's t-test. (C, D) Western blotting of membrane fractions from four groups of mice. Blots probed with antibodies against various receptors and PSD95 showed that the amounts of PSD95 and NR2B, but not other proteins, were higher in APP/PS1 mice than in nonTg mice. Because p-NR2B (NR2B phosphorylated at Tyr1472) did not differ among the four groups, the p-NR2B–to–total NR2B ratio in APP/PS1 mice was lower than that in nonTg mice. However, this difference between saline-treated nonTg and APP/PS1 mice was diminished by PTX treatment. Saline-treated nonTg mice vs. APP/PS1 mice: PSD95, *p = 0.00873; NR2B, *p = 0.00910; p-NR2B/NR2B, *p = 0.00871; two-tailed Student's t-test. Repeated measures two-way ANOVA also showed a significant difference in NR2B levels (F[1,4] = 14.32, *p = 0.0194) but not in PSD95 levels (F[1,4] = 5.27, p = 0.0834). Saline-treated APP/PS1 mice vs. PTX-treated APP/PS1 mice: NR2B, *p = 0.0383; p-NR2B/NR2B, *p = 0.0114; GABA_A_ receptor α1 subunit, not significant p = 0.0810; PSD95, not significant p = 0.4304; two-tailed Student's t-test. Repeated measures two-way ANOVA also showed a significant difference in NR2B levels (F[1,4] = 7.72, *p = 0.0499) but not in PSD95 levels (F[1,4] = 0.31, p = 0.6070).

We were next interested in determining at the protein level how the memory deficits in APP/PS1 mice that are linked to enhanced GABA_A_ receptor-mediated inhibition were improved by PTX treatment. The amount of GABA_A_ receptor α1 subunit in membrane fractions derived from brain homogenates of mice in drug-treatment experiments (Figure 4) was assessed by Western blot. Saline-treated APP/PS1 mice showed stronger GABA_A_ α1 immunoreactivity than saline-treated nonTg mice (Figure 5A). This difference between APP/PS1 and nonTg mice was absent when the mice received PTX treatments (Figure 5A and 5B). These results suggest that enhanced GABA_A_ receptor-mediated inhibition and its associated memory decline in adult APP/PS1 mice might be due to an elevated amount of GABA_A_ receptor α1 subunit, which has also been reported in aged rat hippocampus [18]–[21].

To investigate what might have caused the increased GABA_A_ receptor-mediated inhibition in adult APP/PS1 mice, we further examined changes in the levels of other proteins involved in synaptic plasticity (Figure 5C). Because the immunoreactivity of some antibodies on these samples was somewhat variable, we calculated the averages of immunofluorescent intensities from three blots by normalizing band intensities with those obtained from samples of saline-treated nonTg mice in each blot. Among the five antibodies that we tested, immunoreactivity against PSD95 and NR2B were significantly stronger for samples from saline-treated APP/PS1 mice compared to those from saline-treated nonTg mice (Figure 5D). Because the phosphorylation of NR2B at Tyr1472 stabilizes NMDA receptors on the cell surface [28], making them functionally active, we also tested an antibody against Tyr1472-phosphorylated NR2B (p-NR2B). There was no significant difference among the groups. As a result, the ratio of the relative immunoreactivity of p-NR2B to total NR2B was lower in APP/PS1 mice compared to nonTg mice. As with the case of the GABA_A_ receptor α1 subunit, these differences between APP/PS1 and nonTg mice in the levels of PSD95 and p-NR2B/NR2B also diminished upon PTX treatment through yet unknown mechanisms. Because a statistically significant difference in the levels of PSD95 and GABA_A_ receptor α1 was not detected between saline- and PTX-treated APP/PS1 mice, the comparable levels of these proteins in nonTg and APP/PS1 mice after PTX treatment indicate that there might be some insignificant increase in these proteins in PTX-treated nonTg mice. In the case of NR2B, there was a significant decrease in APP/PS1 mice by PTX treatment in comparison with saline treatment (*p = 0.0383; two-tailed Student's t-test and *p = 0.0499; repeated measures two-way ANOVA), while there was no increase in nonTg mice. Together with our electrophysiological data showing that adult APP/PS1 mice expressed greater LTP than adult nonTg mice after blocking GABA_A_ receptor-mediated inhibition, the abnormally high levels of PSD95 and NR2B potentially indicate that synaptic excitation in adult APP/PS1 mice is overly enhanced, which in turn could lead to a compensatory upregulation of GABA_A_ receptor-mediated inhibition through the activation of homeostatic mechanisms. Although we still do not know exactly how this excitation/inhibition abnormality was created in APP/PS1 mice, their memory deficits, which were linked with a reduction in LTP and induced by enhanced GABA_A_ receptor-mediated inhibition, were improved by PTX treatment, presumably through modifying the levels of proteins involved in synaptic plasticity.

## Discussion

Memory decline in both adult APP/PS1 and old nonTg mice was commonly accompanied by reduced LTP expression that was linked with excessive inhibition via GABA_A_ receptors. On the basis of these findings, we hypothesized that memory decline associated with aging was accelerated in APP/PS1 mice through enhanced GABA_A_ receptor-mediated inhibition resulting from the effects of gene product(s) of early-onset AD such as Aß. In other words, Aß may facilitate aging-associated memory decline through upregulation of GABA-mediated inhibition, even though what mimics the actions of Aß in aging remains to be determined.

A previous report suggested that aberrant excitation causes compensatory enhancement of inhibition by circuit remodeling in hAPP mice [29]. Similarly, in both adult APP/PS1 and old nonTg mice, a greater increase in LTP expression due to the addition of bicuculline compared to the lower increase in LTP in adult nonTg mice suggests abnormal enhancement of both excitation and inhibition. The difference in the efficacy of bicuculline on LTP expression between hAPP and APP/PS1 mice may be attributed to consequences relating to the PS1 mutant gene, because mutant PS1 single transgenic and knock-in mice have been reported to have greater LTP expression and greater vulnerability to excitotoxicity [30]–[34]. Several PS1 mutations are associated with enhanced ryanodine receptor-mediated calcium release through increased receptor expression [35]. Aß renders neurons more vulnerable to excitotoxic insults through destabilization of calcium homeostasis [7]. Increased levels of both PSD95 and NR2B in the brains of adult APP/PS1 mice, which might be generated during homeostasis as Aß downregulates PSD95 and NR2B [36], [37], suggests that the synapses of these mice have the potential to be overly excited. Aß oligomers have also been suggested to form calcium channels by themselves [38]. Excitotoxic insults caused by both excess glutamate release and activation of glutamate receptors are also known to occur with aging [6]. The density of voltage-gated calcium channels coupled with potassium currents is known to increase with age [39]. PSD95 levels are higher in learning-impaired aged rats than in unimpaired aged rats [40]. Age-related increases in GABA_A_ receptor levels has also been reported in the hippocampus of rodents [18]–[21]. Although Aß, mutant PS1, or aging can differentially trigger overexcitation, the outcome (i.e., smaller LTP) of the compensatory upregulation of GABA_A_ receptor-mediated inhibition was commonly linked with memory decline in mice.

We confirmed the reversibility of this abnormality by treating adult APP/PS1 mice with a non-epileptic dose of PTX for 10 days. Modifications of the amounts of GABA_A_ α1, PSD95, and p-NR2B/NR2B as well as cognitive functions caused by the PTX treatment indicated that memory decline in adult APP/PS1 mice resulted, at least in part, from changes in synaptic protein levels through homeostatic mechanisms [41]. Since the protein levels were examined only once after treatment, we could not determine whether the alterations in protein levels were transitory or long-lasting phenomena. This important issue is worth examining in the near future. In the present experiments, the effects were detected in the brains at least one month after the treatment. This is consistent with the effects on the synaptic plasticity and cognitive functions of Ts65Dn mice, a model of Down syndrome, which persisted for a few months after similar treatment with GABA_A_ receptor antagonists [23]. Although we did not test how PTX treatment affects old nonTg mice, long-term treatment of aged rats with subconvulsive doses of pentylenetetrazole, another noncompetitive GABA_A_ receptor antagonist, also improves the cognitive functions of these rats [42], lending support for a common GABA_A_ receptor-mediated mechanism underlying cognitive decline induced by aging and by FAD-liked mutant gene products. During aging, the brain faces progressive challenges and compensates them through the activation of homeostatic mechanisms [43]. Here, we propose that upregulated GABA_A_-receptor mediated inhibition underlies one of the homeostatic mechanisms that are activated by various insults accumulating in the brain during aging such as Aß and that over time compromise synaptic plasticity, neural circuitry, and cognitive functions.

The purpose of PTX treatments in adult APP/PS1 mice was to normalize GABA_A_ receptor-mediated inhibition by maintaining balance between inhibition and excitation to the extent of enhancing LTP and memory. We certainly recognize the risk of excitotoxicity via calcium overload [6]–[8], [35], [38], [39], [44] and that of epilepsy [29] if GABA inhibition is overly and acutely inhibited. Simultaneously targeting excitotoxic insults of various sources hopefully reduces these risks and more directly prevents the initiation of excitation/inhibition abnormalities, as clinically proven by the symptomatic efficacies of a NMDA receptor antagonist on AD patients [45]. The dose of PTX used in this study is non-convulsive but is known to generate anxiety in conflict situations [46]. To see if such an anxiogenic effect was produced in our PTX-treated mice, we analyzed novel object exploration (NOE) from data collected during the NOR experiments (Figure S1 and S2). In nonTg mice, PTX-treated mice, on average, showed less preference for the “objects field” during the training session than during the habituation session, whereas saline-treated nonTg mice showed more preference. Avoidance of objects placed in a familiar field is considered to be a measure of anxiety levels [47]. Thus, as expected from a known anxiogenic effect of PTX treatment [46], this tendency indicates that our PTX-treatment regimen may enhance anxiety levels in nonTg mice even though we failed to find a statistical difference between PTX- and saline- treated groups. This tendency was not even observed in APP/PS1 mice. The question is whether enhanced anxiety or fear can improve cognitive function in these mice. Our results showed that PTX treatment did not change object recognition memory in nonTg mice but enhanced object recognition memory in APP/PS1 mice (Figure 4B). Thus, the results provided no evidence to support the premise that anxiety alone promotes cognitive function. Because the MWM test is itself stressful to mice, it is even more difficult to clearly determine to what extent drug treatment and stress response influenced emotional factors, including anxiety, and to determine their impact on spatial reference memory. Memory and emotion are inseparable and interdependent brain functions [48]. Thus, improving one will directly affect, or be affected by, the other, an observation that may point to an alternative therapeutic approach for AD.

## Materials and Methods

### Animals

We used 9–15-month-old male hemizygous APP/PS1 mice derived from a line that doubly expresses human APP carrying Swedish familial AD-linked mutations (K670N/M671L) and human PS1 encoding the exon 9 deletion mutation, each driven by its own mouse prion protein promoter element [10]. Transgenic (Tg) mice (B6C3-Tg(APP695)85Dbo Tg(PSEN1)85Dbo) were originally purchased from the Jackson Laboratory. This transgenic line had been crossed for 4–6 generations onto the C57BL/6J Jcl (CLEA) background. All of the mice used in this study were males of the C57BL/6J background strain. To examine age-related changes in cognitive function and synaptic plasticity, we classified mice as either “adult” (9–15 months) or “old” (19–25 months). The number and age range (mean±S.D.) of mice used in each experiment and whether wild-type controls were nonTg mice from single or multiple transgenic lines are indicated in the figure legends. All experiments were performed according to procedures approved by the Animal Care and Use Committee of the Institute of Physical and Chemical Research (RIKEN).

### Morris water maze test

To assess place learning and memory performance of mice, we used a cylindrical test apparatus (1 m in diameter) and task fashioned after the Morris water maze. The water was maintained at 24°C, and the maze was surrounded by landmark objects. A slightly submerged transparent platform to which the mice could escape was hidden from view by making the water opaque with a white bio-safe material. The position of the platform was fixed during a 60-sec test period.

Mouse behavior during the water maze test was monitored by a CCD camera mounted overhead; digital data of real-time images were recorded to a PC using the public domain NIH Image program (developed at The National Institute of Mental Health and available on the Internet at http://rsb.info.nih.gov/nih-image/). Images were sampled at 2 Hz. Data was analyzed using customized software based on Matlab (version 7.2, Mathworks Co. Ltd., MA) with image analysis tool box (Mathworks Co. Ltd.). During testing, the sequential position of the mouse was determined in each video frame, and the swimming speed, distance from platform, and latency to reach the platform were calculated. To assess learning, for each mouse we measured the distance between the mouse and the platform every 0.5 sec until the mouse reached the platform. Then, we integrated these distances between the mouse and the platform during each trial. This “integrated distance” value represents the error score, which is used here as a measure of learning performance [49]. The error score was slightly modified from “search error” or “behavioral index,” two parameters described previously [5], [15]. To assess the accuracy of memory, we also calculated the probe score by dividing the error score derived during the probe test by the total number of recorded frames (a frame/0.5 sec×60 sec = 120 frames).

For each learning trial, the mouse was gently placed on the water surface close to the cylinder wall in each quadrant at random (except the target quadrant where the platform was located). The entry quadrant for each trial was always different from that of the prior trial. The mouse was allowed to swim freely for a 60-sec test period. When the mouse did not escape to the submerged platform within this test period, we gently navigated it to the platform by hand and made it stay there for 20 sec. For each mouse, we carried out three learning trials per day for nine successive days. Four probe trials were given on the tenth day, in which the platform was removed from the maze in the mouse's absence. The mouse was introduced into the maze as before and allowed to search for the missing platform for 60 sec. The maze surface was logically partitioned into quadrants for analysis, and the percentage of time that the mouse spent searching in each quadrant was calculated. This percentage was used as an index of memory performance, the logic being that if the mouse remembered where the platform was during training, it would spend a disproportionate percentage of its swimming time during the probe trial searching in the quadrant where the platform was located during the learning trials. Statistical analyses were conducted using PRISM4 (GraphPad Software Inc., CA). Data were analyzed using the Friedman test or repeated measures two-way ANOVA, unless noted otherwise.

### Novel object recognition test

The NOR task is based on the innate tendency of rodents to differentially explore novel objects over familiar ones [23], [47]. Mice were trained and tested twice, with each experimental session separated by 3 weeks; they were serially presented with new object sets. In this scheme, each mouse is considered to be a naive subject, and each performance is considered to be an independent observation [50].

Saline- or PTX-treated adult nonTg and adult APP/PS1 mice were given an opportunity to habituate to a new cage (17 cm×28 cm×12 cm). After 15 min in this cage, mice were exposed to two identical objects during a 15-min training session. Objects were made from either metal or plastic (LEGO blocks) and had various color schemes. Four groups of mice were tested with these objects pseudo-randomly to make sure that, within an experiment, each group of mice was tested with the same type of object. The pairings of objects are shown in Figure S1. All objects were generally consistent in height and volume. They were positioned in two corners of the experimental field (Figure S1). A 15-min testing session was conducted right after training. Here, the mice were presented with two objects—the object they had explored in the training session and a new object. Novel object recognition memory was operationally defined as the proportion of time animals spent investigating the novel object (or an identical object presented during the training session) and measured in terms of a Preference Index (P. I. = (Novel Object Exploration Time/Total Exploration Time)×100), where exploration constituted any investigative behavior (i.e., head orientation, sniffing) or deliberate contact that occurred with each object. To control for odor cues, the experimental cage was changed for each mouse, and the objects were thoroughly cleaned with 90% ethanol, dried, and ventilated for a few minutes between mice.

### *In Vitro* fEPSP Recordings

Hippocampal slices (350 µm thick; horizontally sectioned) were prepared in ice-cold aCSF (in mM, 124 NaCl; 2.5 KCl; 2 CaCl_2_; 2 MgSO_4_; 1.25 NaH_2_PO_4_; 26 NaHCO_3_; and 10 glucose) continuously bubbled with 95% O_2_/5% CO_2_. Slices were allowed to recover in aCSF at room temperature for >1 hour prior to recording, after which they were maintained at 29–30°C in the same oxygenated conditions. Baseline responses were obtained every 10 sec, and 3–6 responses were averaged to obtain a data point. Stimulation intensity was subsequently set to a level that produced a fEPSP slope value of about 40% of the maximum obtained. LTP was induced by delivering a single train of tetani (100 stimuli, 100 Hz) through a bipolar tungsten electrode positioned in the molecular layer of the dentate gyrus. Field potential recordings were obtained with borosilicate glass capillaries filled with 0.5 M NaCl (0.3–0.5 MΩ) situated in the dentate gyrus molecular layer. Slices were bathed in aCSF with or without 20 µM bicuculline.

### Western Blotting

Mouse brains were homogenized in Tris-buffered saline (TBS; 10 mM Tris, 150 mM NaCl [pH 7.4]) containing protease inhibitors (1 µg/ml antipine, 5 µg/ml pepstatin, 5 µg/ml leupeptin, 2 µg/ml aprotinin, and 0.5 µM 4-(2-aminoethyl)benzenesulfonyl fluoride hydrochloride). After centrifugation at 100,000*g* for 20 min at 4°C, the supernatant was collected. The resulting precipitate was re-homogenized in 3 volumes of modified RIPA buffer (50 mM Tris [pH 7.4], 1% NP-40, 0.25% Sodium deoxycholate, 150 mM NaCl, and 1 mM EGTA) and centrifuged at 100,000*g* for 20 min at 4°C. The supernatant was analyzed as the “membrane protein” fraction. After measuring and normalizing the total protein concentration by the Bradford method, samples from this fraction were solubilized in Laemmli sample buffer and subjected to SDS-PAGE. Separated proteins were blotted onto a nitrocellulose membrane. The membrane was incubated with the primary antibody followed by HRP-conjugated secondary antibody. Chemiluminescent detection (ECL, Amersham) was used for visualization. The primary antibodies used were anti-GABA_A_ receptor α1 subunit (Sigma); GluR1 (Chemicon); GluR2 (Chemicon); PSD95 (BD Transduction); NR2A (BD Transduction); NR2B (BD Transduction); and phospho-Tyr1472 NR2B (Chemicon). Quantitation and visual analysis of immunoreactivity were performed with a computer-linked LAS-3000 Bio-Imaging Analyzer System (Fujifilm).

## Supporting Information

Figure S1 Type, pair, and position of objects during novel object recognition (NOR) tests. (A) Four types of metal objects characterized by their shape were paired as shown for the testing session of the first set of NOR tests. The position of a novel object is indicated by the letter X or Y, which corresponds to the corner of the experimental field as shown in the schematic diagram showing a bird's eye view of the test apparatus. Six animals from each group of the drug-treatment experiment were subjected to the six different experimental schemes (Figure 4). (B) For the second set of NOR tests, four types of objects were made from LEGO blocks. Each object was characterized by a combination of two colors and by different shapes. Objects used for the experimental scheme in (A) were replaced with the LEGO objects.
(1.73 MB TIF)

Figure S2 Effect of PTX treatment on the anxiety levels of mice: novel object exploration (NOE). Avoidance of objects placed in a familiar field is considered as a measure of anxiety level [1]. The anxiety level of a mouse was estimated by measuring the avoidance of two novel and two identical objects placed into a familiar environment during the training session of the novel object recognition (NOR) test. Thus, mice used in this NOE experiment are the same cohort used in the NOR test (saline-treated nonTg, saline-treated APP/PS1, PTX-treated nonTg, and PTX-treated APP/PS1). The experimental field was divided into three areas, as schematically represented in Figure S1. The “objects field” represents the area located between the left wall and 2.5 cm just left to the midpoint line. The “center field” represents the area located between 2.5 cm left and just right to the midpoint line. The “objectless field” represents the rest of the experimental field. During the habituation and training sessions, the number of entries into the objects field was counted each time a mouse in the objectless field enters into the objects field by passing through the center field. Thus, to be counted as one entry, a mouse had to move to the objectless field and then enter into the objects field. This calculation scheme was used to avoid counting ambiguous entries. The number of entries during the last 5 min of the habituation session was taken as baseline. The number of entries during the first 5 min of the training session relative to baseline was calculated in order to correct for possible preferences for a particular field. In nonTg mice, PTX-treated group on average showed less preference or more avoidance of the objects field during the training session than the habituation session whereas saline-treated group showed more preference for the objects field although a statistical significance of neither tendency was supported by binomial analysis (p = 0.0537 for the avoidance of the objects field by PTX-treated nonTg mice and p = 0.2256 for the preference of the objects field by saline-treated nonTg mice). In APP/PS1 mice, such a tendency to avoid the objects field was not observed in PTX-treated group. The dotted horizontal line represents chance performance. Data are presented as means with error bars denoting S.E.M. Reference 1. van Gaalen MM, Steckler T (2000) Behavioural analysis of four mouse strains in an anxiety test battery. Behav Brain Res 115: 95–106.
(0.49 MB TIF)
