# Supplementary figures and images for: GABAA Receptor-Mediated Acceleration of Aging-Associated Memory Decline in APP/PS1 Mice and Its Pharmacological Treatment by Picrotoxin
Source: PLoS One. 2008 Aug 21;3(8):e3029. doi: 10.1371/journal.pone.0003029 (PMC2515633; doi:10.1371/journal.pone.0003029)

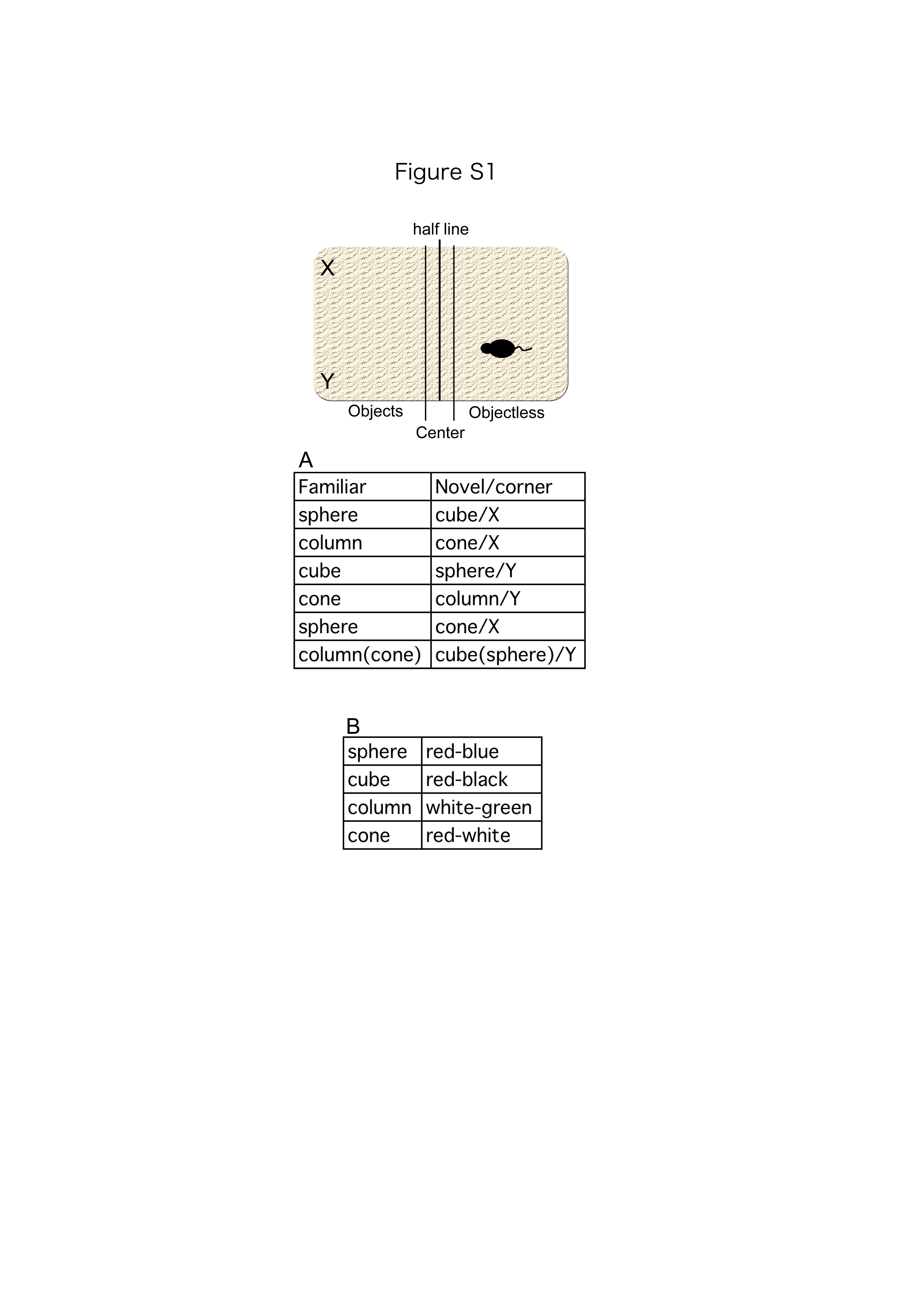

Supplement: Figure S1 — Type, pair, and position of objects during novel object recognition (NOR) tests. (A) Four types of metal objects characterized by their shape were paired as shown for the testing session of the first set of NOR tests. The position of a novel object is indicated by the letter X or Y, which corresponds to the corner of the experimental field as shown in the schematic diagram showing a bird's eye view of the test apparatus. Six animals from each group of the drug-treatment experiment were subjected to the six different experimental schemes (Figure 4). (B) For the second set of NOR tests, four types of objects were made from LEGO blocks. Each object was characterized by a combination of two colors and by different shapes. Objects used for the experimental scheme in (A) were replaced with the LEGO objects. (1.73 MB TIF) [file pone.0003029.s001.tif]

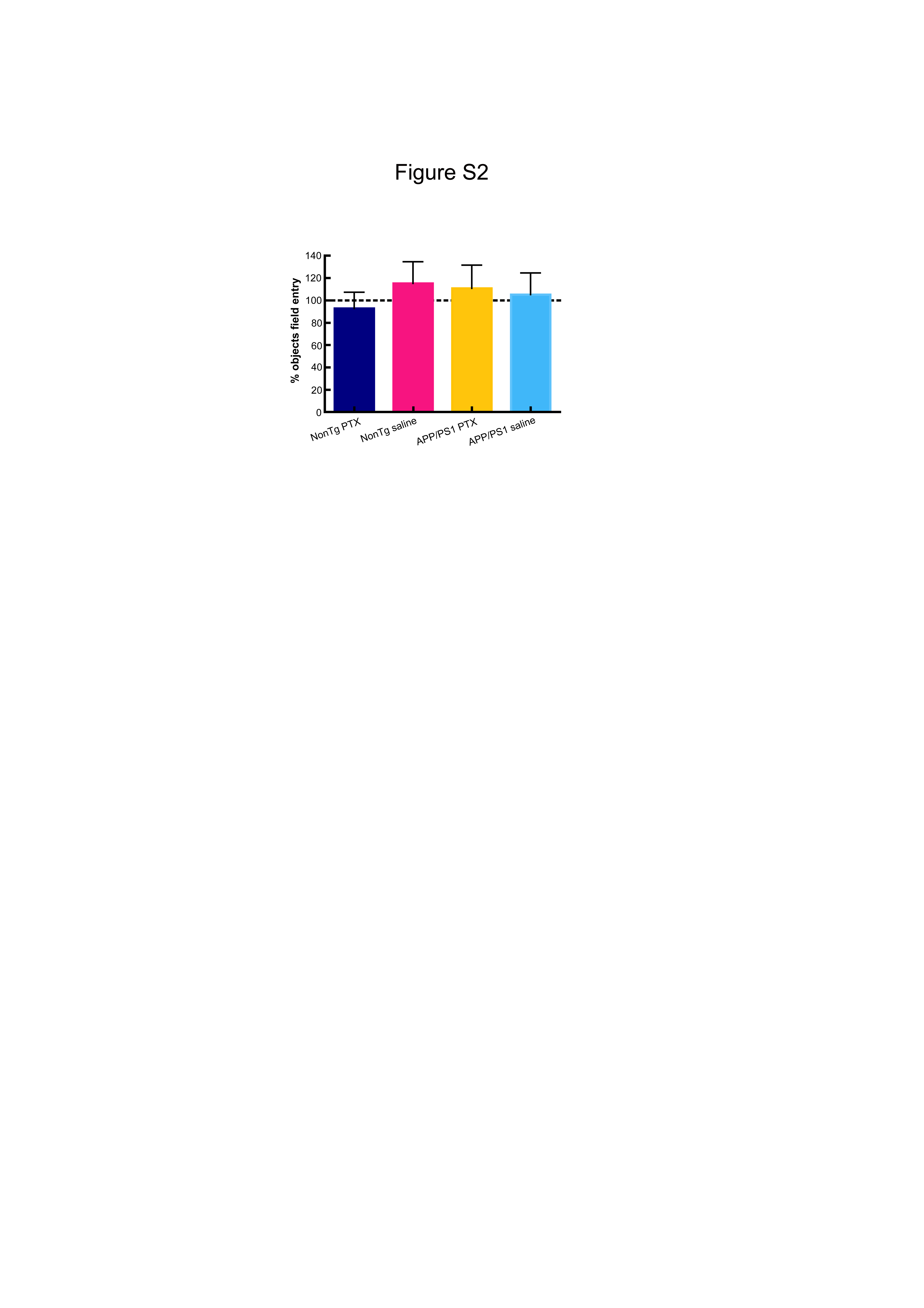

Supplement: Figure S2 — Effect of PTX treatment on the anxiety levels of mice: novel object exploration (NOE). Avoidance of objects placed in a familiar field is considered as a measure of anxiety level [1]. The anxiety level of a mouse was estimated by measuring the avoidance of two novel and two identical objects placed into a familiar environment during the training session of the novel object recognition (NOR) test. Thus, mice used in this NOE experiment are the same cohort used in the NOR test (saline-treated nonTg, saline-treated APP/PS1, PTX-treated nonTg, and PTX-treated APP/PS1). The experimental field was divided into three areas, as schematically represented in Figure S1. The “objects field” represents the area located between the left wall and 2.5 cm just left to the midpoint line. The “center field” represents the area located between 2.5 cm left and just right to the midpoint line. The “objectless field” represents the rest of the experimental field. During the habituation and training sessions, the number of entries into the objects field was counted each time a mouse in the objectless field enters into the objects field by passing through the center field. Thus, to be counted as one entry, a mouse had to move to the objectless field and then enter into the objects field. This calculation scheme was used to avoid counting ambiguous entries. The number of entries during the last 5 min of the habituation session was taken as baseline. The number of entries during the first 5 min of the training session relative to baseline was calculated in order to correct for possible preferences for a particular field. In nonTg mice, PTX-treated group on average showed less preference or more avoidance of the objects field during the training session than the habituation session whereas saline-treated group showed more preference for the objects field although a statistical significance of neither tendency was supported by binomial analysis (p = 0.0537 for the avoidance of the objects field [file pone.0003029.s002.tif]
